# Biodegradable PCL-b-PLA Microspheres with Nanopores Prepared via RAFT Polymerization and UV Photodegradation of Poly(Methyl Vinyl Ketone) Blocks

**DOI:** 10.3390/polym13223964

**Published:** 2021-11-16

**Authors:** Taeyoon Kim, Sorim Lee, Soo-Yong Park, Ildoo Chung

**Affiliations:** 1 Department of Polymer Science and Engineering, Pusan National University, Busan 46241, Korea; xodbs17@skku.edu (T.K.); so173028@pusan.ac.kr (S.L.); dhd369@pusan.ac.kr (S.-Y.P.); 2 Convergence Research Center for Energy and Environmental Sciences, Sungkyunkwan University, Suwon 16419, Korea

**Keywords:** photodegradation, block copolymer, RAFT, MVK, porous polymer

## Abstract

Biodegradable triblock copolymers based on poly(ε-caprolactone) (PCL) and poly(lactic acid) (PLA) were synthesized via ring-opening polymerization of L-lactide followed by reversible addition–fragmentation chain-transfer (RAFT) polymerization of poly(methyl vinyl ketone) (PMVK) as a photodegradable block, and characterized by FT-IR and ^1^H NMR spectroscopy for structural analyses, and by differential scanning calorimetry (DSC) and thermogravimetric analysis (TGA) for their thermal properties. Porous, biodegradable PCL-b-PLA microspheres were fabricated via the oil/water (O/W) emulsion evaporation method, followed by photodegradation of PMVK blocks by UV irradiation. The macro-chain transfer agent (CTA) synthesized by reacting a carboxylic-acid-terminated CTA—S-1-dodecyl-S′-(a,a′-dimethyl-a′′-acetic acid)trithiocarbonate (DDMAT)—with a hydroxyl-terminated PCL-b-PLA block copolymer was used to synthesize well-defined triblock copolymers with methyl vinyl ketone via RAFT polymerization with controlled molecular weights and narrow polydispersity. Gel permeation chromatography traces indicated that the molecular weight of the triblock copolymer decreased with UV irradiation time because of the photodegradation of the PMVK blocks. The morphology of the microspheres before and after UV irradiation was investigated using SEM and videos of three-dimensional confocal laser microscopy, showing a change in their surface texture from smooth to rough, with high porosity owing to the photodegradation of the PMVK blocks to become porous templates.

## 1. Introduction

Biodegradable aliphatic polyesters such as PCL and PLA, endowed with semicrystallinity and biodegradability due to their potentially hydrolyzable ester bonds, have been widely used as scaffolds in tissue engineering, drug delivery systems, packaging, and biomedical applications, because of their controlled biodegradability, biocompatibility, and good physical properties [1,2,3]. It has been reported that, compared to PLA, the degradation period of PCL can range from several months to a few years, and its biodegradability is dependent on its molecular weight, degree of crystallinity, and degradation conditions. Although PCL has been considered for use as a drug delivery matrix, owing to its favorable permeability to drugs and thermal properties, it is known as a relatively biostable polymer compared to PLA, resulting in its limited use for targeted therapeutic applications requiring a controlled degradation rate. A block copolymer composed of L-lactide and ε-caprolactone was selected in this study, with the advantages of both the degradability of PLA and the permeability of PCL to drugs [4,5,6,7]. In particular, branched polymers—including four-armed star biodegradable polymers—have been reported to be more efficient for higher drug loading and controlled drug delivery, due to their suitability for the formation of porous structures [8,9,10].

Considerable research has been focused on the encapsulation of drugs into polymeric carriers, which can provide several advantages over conventional formulations, such as the protection of drugs from metabolism or degradation. Several studies have investigated the effects of nanoparticle shapes and surface morphologies on controlled release as drug matrices for both spherical and non-spherical particles [11]. Spherical polymeric particles have been studied intensively and fabricated via various methods, such as spray-drying and emulsification methods. Various parameters for drug carriers—such as surface, size, and shape—should also be considered [11,12,13,14]. Several research groups have reported that surface morphology and particle shape play important roles in the interactions of particles with cells and tissues in the human body [13,15,16,17,18]. The fabrication of non-spherical particles usually takes place via two main pathways—ab initio synthesis, or post-modification from spherical particles—including a range of techniques such as lithography, microfluidics, and photopolymerization [11,19,20,21]. In recent years, considerable efforts have been made to develop nano/microspheres based on PCL and PLA for the transport and controlled release of various drugs [5]. Porous polymers can be designed to confer several advantages, including high surface area, well-defined porosity, and easy processability compared to inorganic materials [12]. This is due to the tailored design and controllable porosity of polymers, achieved by incorporating chemical functionalities into porous structures [22,23,24,25]. Numerous studies have been focused on the development of porous polymer-based microspheres, using techniques such as direct templating, block copolymer self-assembly, direct synthesis, high-internal-phase emulsion polymerization, and interfacial polymerization [26,27,28,29,30,31]. For example, self-assembled block copolymers (BCPs), as a sacrificial component, could be regarded as hybrid polymers consisting of two or more chemically immiscible polymers that are linked together covalently. Although phase separation is generated by self-assembly because of incompatibility between these segments, minimizing surface energy, the separation is restricted owing to segment chain connectivity [12,32]. Among the aforementioned technologies that allow for the controlled fabrication of porous polymers, direct templating is a useful method, because of the diverse templates and well-defined porous structures created by the removal of pore templates through the use of solvents, enzymes, or inorganic acids [12,14,33,34]. However, to the best of our knowledge, our previous report is the only one focusing on the fabrication of porous microsphere polymers using UV light [35], which is potentially a more advantageous method, as the porous microspheres could be formed without using additional templates. The resulting porous polymers could be used for gas storage, separation materials, encapsulation agents for the controlled release of drugs and catalysts or supports for immobilizers and cell scaffolds [36,37,38,39,40,41,42,43,44].

Poly(vinyl ketones), photodegraded by UV irradiation, have been used not only as components for packing materials and agricultural films, but also as functional materials in applications such as imaging, microfabrication, and sensors [45,46,47,48]. Poly(methyl vinyl ketone) (PMVK) can be degraded to its low-molecular-weight fragments by photochemical reactions through either radical or non-radical reactions, which are known as Norrish Type I and II routes [45,48]. Over the past few decades, considerable research has been conducted to develop controlled radical polymerizations (CRPs)—such as reversible deactivation radical polymerization (RDRP), reversible addition–fragmentation chain-transfer polymerization (RAFT), atom transfer radical polymerization (ATRP), and nitroxide-mediated polymerization (NMP)—to synthesize well-defined polymers with controlled structures, predetermined molecular weights, and narrow polydispersity [49]. This study aimed to overcome the limitations of conventional free radical polymerization (CFRP) resulting from irreversible bimolecular termination between propagating radical species [50,51,52,53,54,55]. Unlike NMP and ATRP, RAFT controls polymerization based on the principle of degenerative reversible chain transfer via deactivation−activation equilibrium [56,57]. Although ATRP has been proven to be a versatile tool for the synthesis of well-defined polymers, and has been applied to a wide variety of functional monomers, unfavorable complexation between MVK monomers and metal catalysts is a major factor in the depressed polymerization rate and subsequent failure of ATRP in the polymerization of MVK [58].

In this study, we achieved the successful synthesis of triblock copolymers from the ring-opening polymerization of PLA onto PCL diol, followed by RAFT polymerization of PMVK and, finally, the fabrication of biodegradable porous microspheres via a direct templating methodology resulting from photodegradation of the PMVK block by UV irradiation. A PCL-b-PLA block copolymer was first synthesized via ring-opening polymerization and used as a macro-CTA after reacting with S-1-dodecyl-S’-(a,a’-dimethyl-a’’-acetic acid)trithiocarbonate (DDMAT) for RAFT polymerization of PMVK to obtain PCL-b-PLA-b-PMVK triblock copolymers. The synthesized triblock copolymers were characterized by FT-IR and ^1^H NMR spectroscopy. Gel-permeation chromatography (GPC) was used to evaluate the polymer’s molecular weight and distribution, and to monitor the photodegradability of the triblock copolymers. The microspheres were spherical, with smooth surfaces, before UV irradiation; however, after UV irradiation, the surfaces became rough, with highly porous structures, owing to the photodegradation of the PMVK blocks. The porosity and shape of the microspheres were found to depend on both the PMVK content and the microsphere size.

## 2. Materials and Methods

### 2.1. Materials

Linear and 4-armed star poly(ε-caprolactone) with molecular weights of 4 K and 1 K, respectively, were kindly received from Perstorp (Malmö, Sweden) and used without purification. Methyl vinyl ketone purchased from Sigma-Aldrich Co. (Saint Louis, USA) was passed through a column filled with basic alumina and dried by distillation over CaH_2_ prior to use. Stannous 2-ethylhexanoate (Sn(Oct)_2_) was purchased from Sigma-Aldrich Co. (USA) and used without purification. L-Lactide was purchased from Tokyo Chemical Industry Co. (Tokyo, Japan) and used without purification. 4-Dimethylaminopyridine (DMAP) and N,N’-dicyclohexylcarbodiimide (DCC) were purchased from Alfa Aesar Co. (Haverhill, USA) and used without purification. 1,4-Dioxane and dichloromethane (DCM) were purchased from DaeJung Chemicals & Metals Co. (Siheung, Korea) and purified by distillation over CaH_2_. S-1-Dodecyl-S’-(a,a’-dimethyl-a’’-acetic acid)trithiocarbonate (DDMAT) was synthesized according to a method similar to that used in our previous work [35]. All other chemicals were of analytical grade and used without further purification.

### 2.2. Characterization

^1^H and ^13^C NMR spectra were recorded in CDCl_3_ using a Varian 400 spectrometer (Agilent Technologies, Santa Clara, USA). FT-IR spectra were obtained at a resolution of 4 cm^−1^ with an Agilent Cary-640 spectrometer (Agilent Technologies, Santa Clara, USA) in the wavenumber range of 4000~400 cm^−1^. The powder samples were incorporated into KBr pellets for IR absorption analysis. Thermal degradation was examined using a TA Q50 thermogravimetric analyzer (TGA, TA Instruments, New Castle, USA) with a heating rate of 10 °C/min under a nitrogen atmosphere. Melting points were recorded from -50 to 100 °C in the second cycle to remove thermal hysteresis in a TA Q20 differential scanning calorimeter (DSC). The molecular weight and polydispersity index were measured via gel-permeation chromatography (GPC) with a refractive index detector. Tetrahydrofuran (THF) was used as the eluent (flow rate of 1 mL/min), and the molecular weight was calibrated using polystyrene standards. Field-emission scanning electron microscopy (FE-SEM) observations were performed using a Zeiss SUPRA 25 (Carl Zeiss, Oberkochen, Germany). Particle size measurements were performed using a Beckman Coulter LS 13 320 laser-diffraction particle size analyzer (Beckman Coulter, Brea, USA) under aqueous liquid mode, and dynamic light scattering (DLS) with a Malvern S90 (Malvern Panalytical, Malvern, UK), using water as a solvent. Multiphoton confocal microscopy images were acquired using a ZEISS LSM 780 configuration 16 NLO microscope (Carl Zeiss, Oberkochen, Germany), with Nile red as the fluorescent agent.

### 2.3. Synthesis of Poly(ε-caprolactone) (PCL)-b-poly(lactic Acid) (PLA) Diblock Copolymers

**Scheme 1:**
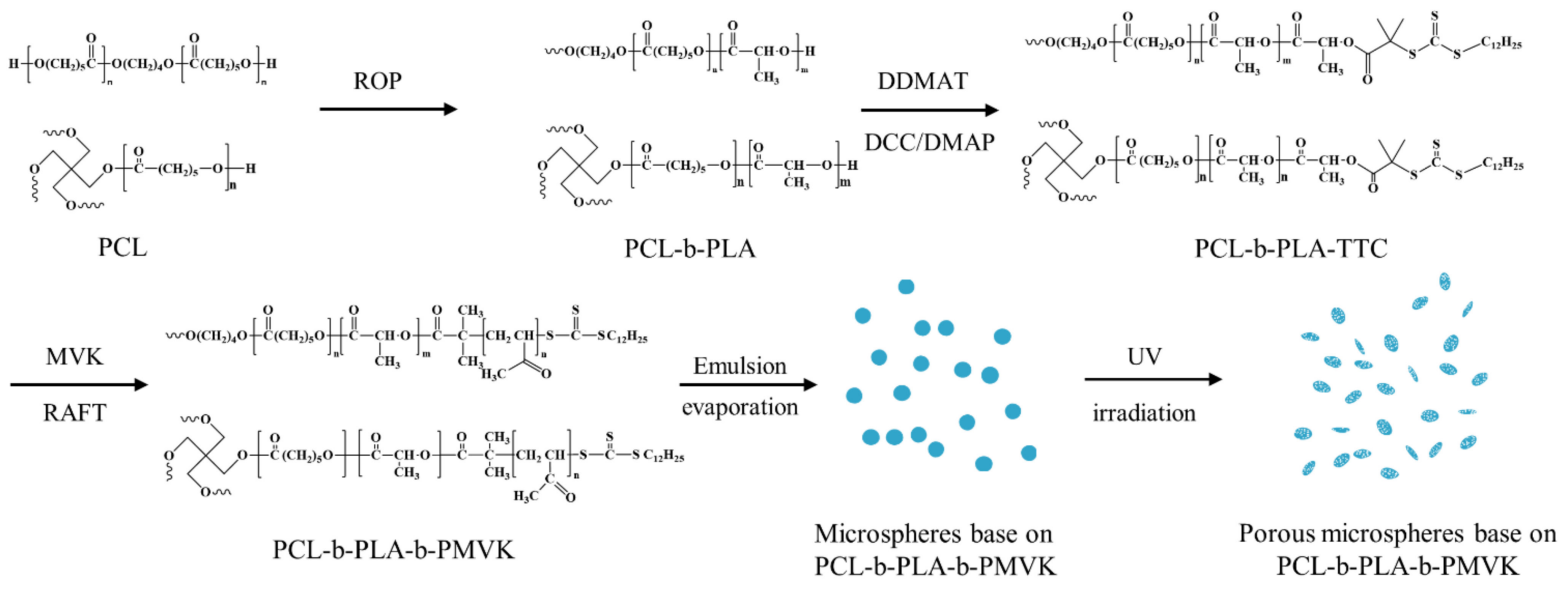
Fabrication of porous microspheres based on PCL-b-PLA-b-PMVK.

As shown in Scheme 1, PCL-b-PLA diblock copolymers were synthesized via ring-opening polymerization of L-lactide in the presence of poly(ε-caprolactone) diol, using Sn(Oct)_2_ as a catalyst. In a representative experiment, PCL (10 g, 2.5 mmol), L-lactide (10 g, 69.4 mmol), and Sn(Oct)_2_ (70 mg, 0.29 mmol) were added to a Schlenk flask with a magnetic stir bar, and dissolved completely at 95 °C, after which the flask was degassed by reduced pressure. After the flask was backfilled with nitrogen, polymerization was performed under a nitrogen atmosphere at 130 °C for 6 h. After the reaction mixture was diluted with 20 mL of chloroform, PCL-b-PLA diblock copolymers were recovered by precipitation from methanol, followed by drying in a vacuum oven at ambient temperature. ^1^H NMR in CDCl_3_ {δ, ppm}: 5.13 (^1^H, methine proton of PLA), 1.57 (3H, methyl protons of PLA), 1.18–1.19 (6H, methylene protons of PCL), 2.28 (2H, -COOCH_2_- of PCL), 4.04 (2H, -CH_2_COO- of PCL), 4.33 (1H, ω-methine proton of PLA), 1.48 (3H, ω-methyl proton of PLA; FT-IR in NaCl{ ν, cm^-1^}; 3000~3500(w; νs(O-H stretching of ω-hydroxyl group)), 1758 (s; νs(C=O from PCL)), 1733 (s; νs(C=O from PLA)), 1456 (w), 1420 (m), 1359 (m), 1388 (w, νb(C-H from PCL, PLA)); *M*n, NMR = 8100 g mol^−1^, *M*n, SEC = 15,900 g mol^−1^, *Đ* = 1.21.

### 2.4. Synthesis of CTA Terminated PCL-b-PLA Diblock Copolymers (Macro-CTA)

To a solution of PCL-b-PLA diblock copolymer (8 g, 1.02 mmol), DDMAT (1.49 g, 4.08 mmol) in 40 mL of dichloromethane (DCM), N,N’-dicyclohexylcarbodiimide (DCC) (0.84 g, 4.08 mmol), and 4-dimethylaminopyridine (DMAP) (0.25 g, 2.04 mmol) in 20 mL of DCM were added dropwise for 1 h. The reaction mixture was then allowed to react overnight at room temperature, with continuous stirring. After the reaction was completed, the N,N’-dicyclohexylurea formed was filtered off, and the filtrate was precipitated in methanol. The precipitate was collected by filtration, washed several times with methanol and, finally, dried to a constant weight under vacuum at ambient temperature to obtain pure PCL-b-PLA diblock macro-CTA. ^1^H NMR in CDCl_3_ {δ, ppm}: 0.8 (2H, -SCH_2_- of DDMAT), (3H, -CH_3_- of DDMAT), 1.24 (6H, -C(CH_3_)_2_- of DDMAT), 5.13 (^1^H, methine proton of PLA), 1.57 (3H, methyl protons of PLA), 1.18–1.19 (6H, methylene protons of PCL), 2.28 (2H, -COOCH_2_- of PCL), 4.04 (2H, -CH_2_COO- of PCL); *M*n, NMR = 8900 g mol^−1^, *M*n, SEC = 20,100 g mol^−1^, *Đ* = 1.24.

### 2.5. Synthesis of PCL-b-PLA-b-PMVK Triblock Copolymer by RAFT Polymerization

The solution of PCL-b-PLA macro-CTA (3 g, 0.353 mmol), azobisisobutyronitrile (AIBN) (0.0058 g, 0.0353 mmol), and MVK (4.41 mL, 62.92 mmol) in 1,4-dioxane was added to a 100 mL Schlenk flask, and the reaction mixture was degassed via three freeze–pump–thaw cycles to remove oxygen. After the flask was backfilled with nitrogen, polymerization was carried out at 70 °C for 8 h. The solution was diluted with 1,4-dioxane to terminate the polymerization, and subsequently precipitated in methanol with vigorous stirring. The precipitate formed was collected and dried under vacuum to obtain a pure PCL-b-PLA-b-PMVK triblock copolymer. ^1^H NMR in CDCl_3_ {δ, ppm}: 1.70~1.56 (4H, CH_2_CH-, polymer main chain of PMVK), 2.1~2.50 (4H, CH_2_CH-, polymer main chain of PMVK), 1.24 (s, 1H), 0.8 (2H, -SCH_2_- of DDMAT), (3H, -CH_3_- of DDMAT), 1.24 (6H, -C(CH_3_)_2_- of DDMAT), 5.13 (1H, methine proton of PLA), 1.57 (3H, methyl protons of PLA), 1.18–1.19 (6H, methylene protons of PCL), 2.28 (2H, -COOCH_2_- of PCL), 4.04 (2H, -CH_2_COO- of PCL); FT-IR in NaCl{ ν, cm^−1^}; 3000~3500(w; νs(O-H stretching of ω-hydroxyl group)), 1758 (s; νs(C=O from PCL)), 1733 (s; νs(C=O from PLA)), 1712 (s; νs(C=O from PMVK)), 1456 (w), 1420 (m), 1359 (m), 1388 (w, νb(C-H from PCL, PLA)); *M*n, NMR = 12,700 g mol^−1^ *M*n, SEC = 26,200 g mol^−1^, *Đ* = 1.17.

### 2.6. Fabrication of Porous Microspheres

Triblock copolymers (PCL-b-PLA-b-PMVK, 0.85 g) dissolved in DCM (2.5 mL) were added dropwise to 50 mL of polyvinyl alcohol (PVA) aqueous solution (10%, *w/v*) for 10 min. After the reaction was allowed to continue for an additional 8 h with stirring of 600 rpm, the emulsion phase formed was centrifuged at 12,000 rpm for 10 min, and the supernatant was removed. The obtained microspheres were redispersed in ethanol and centrifuged again. After five cycles of centrifugation and removal of the supernatant, the microspheres were dispersed in water and then UV-irradiated at a wavelength of 312 nm and various UV exposure times. After the UV irradiation was completed, the solution was centrifuged at 12,000 rpm for 10 min, and the supernatant was removed once more. Finally, the sedimented microspheres were dispersed in water and lyophilized for 2 days to obtain porous microspheres.

## 3. Results and Discussion

### 3.1. Characterization of Di and Triblock PCL-b-PLA Copolymers

**Figure 1:**
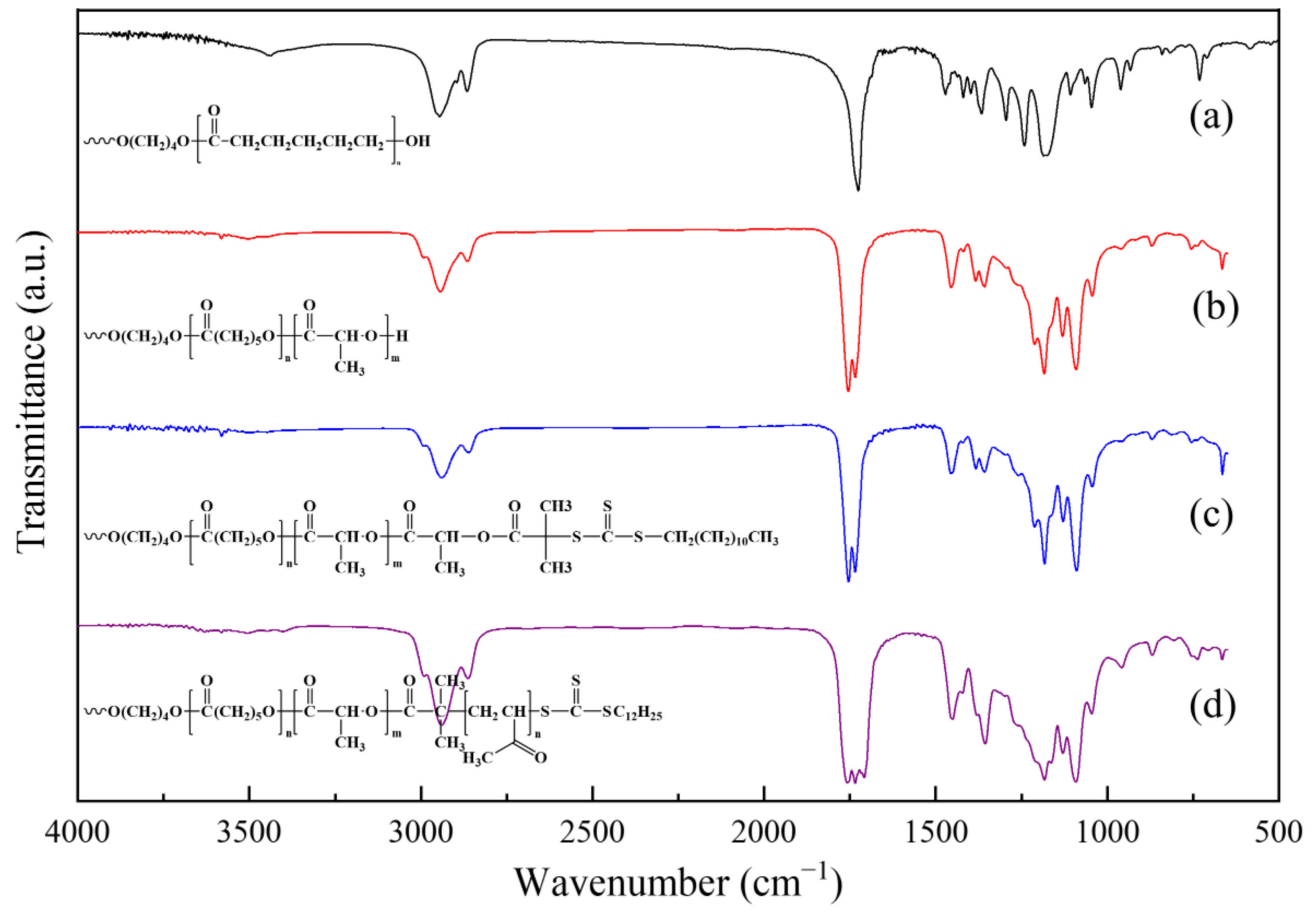
FT-IR spectra of (**a**) PCL, (**b**) PCL-b-PLA, (**c**) PCL-b-PLA-TTC, and (**d**) PCL-b-PLA-b-PMVK.

Various characterization techniques—including FTIR, ^1^H NMR, GPC, TGA, DSC, FE-SEM, and confocal microscopy—were used to identify the structural and morphological changes of block copolymers and their porous microspheres. FT-IR analysis was used to investigate the chemical changes caused by the ring-opening polymerization of L-lactide initiated by poly(ε-caprolactone). In the FT-IR spectrum of the PCL-b-PLA block copolymer, as shown in Figure 1b, two types of carbonyl groups arose from both PCL and PLA, exhibiting strong FT-IR peaks at approximately 1758 and 1733 cm^−1^, respectively.

**Figure 2:**
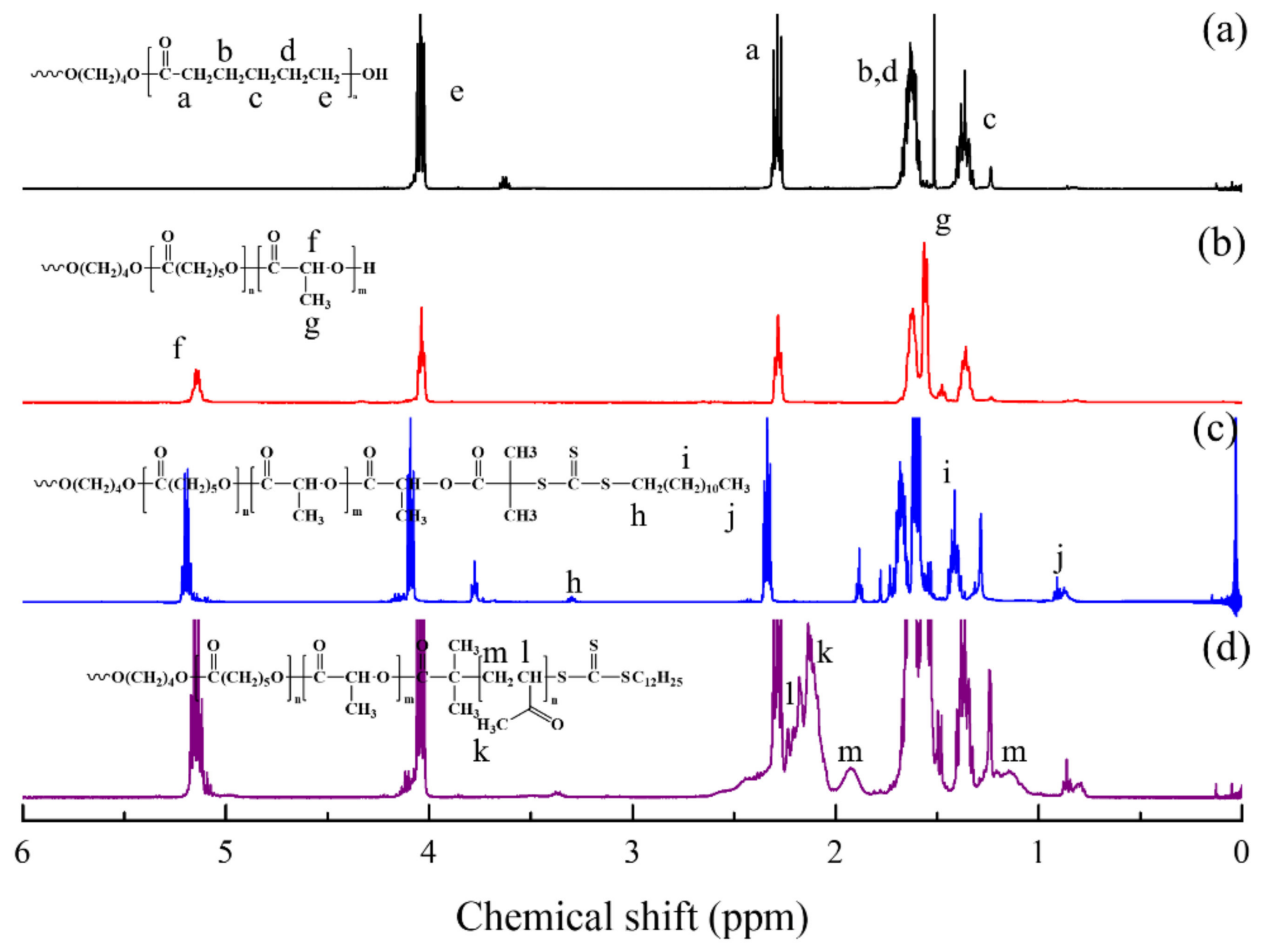
^1^H NMR spectra of (**a**) PCL, (**b**) PCL-b-PLA, (**c**) PCL-b-PLA-TTC, and (**d**) PCL-b-PLA-b-PMVK.

**Figure 3:**
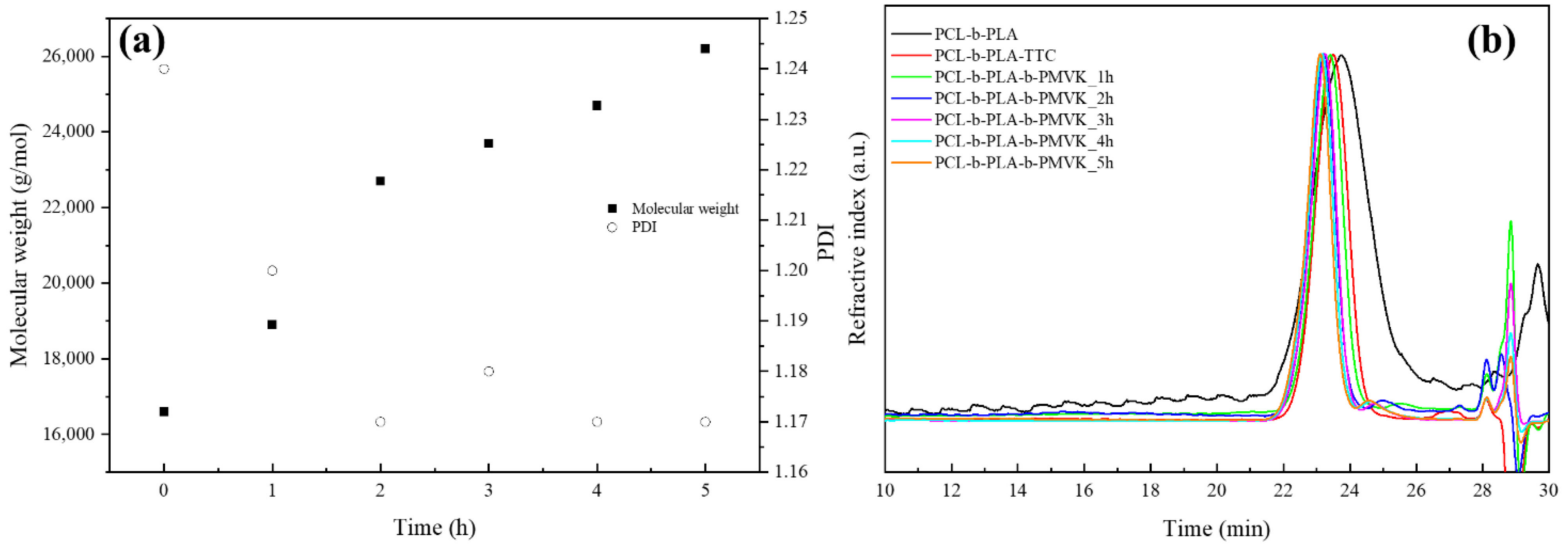
Molecular growth of (**a**) PCL-b-PLA-b-PMVK, and (**b**) its GPC diagrams.

In the ^1^H NMR spectra, the methine peak of PLA was shifted slightly downfield, from 5.08 to 5.15 ppm, after polymerization. The methyl peak of the PLA block was also confirmed at 1.56 ppm in the ^1^H NMR spectrum of PCL-b-PLA (Figure 2b). As shown in Table 1 and Figure 3, GPC analysis indicated that the molecular weight of PCL-b-PLA block copolymers was found to be 7500–15,900, maintaining narrow polydispersity and symmetrical monomodal GPC curves. DDMAT is a particularly suitable CTA for RAFT polymerization, since it is commercially available, possesses a carboxylic reactive end-group and, moreover, can be used to polymerize styrenes, acrylates, acrylamides, methacrylates, methacrylamides, and vinyl ketones [59]. To confer RAFT-polymerizable functionality at the chain-end of PCL-b-PLA, trithiocarbonate (TTC) was incorporated into PCL-b-PLA by reacting the hydroxyl group of PCL-b-PLA with the carboxylic acid of DDMAT.

In the ^1^H NMR spectrum of PCL-b-PLA-TTC (Figure 2c), protons of the methylene group (-S-CH_2_-) of TTC at 3.25 ppm were observed, along with disappearance of those of the ω-methine group (-CH-OH) of PLA at 4.04 ppm. The trithiocarbonate group of TTC was also observed at 225 ppm in the ^13^C NMR spectrum (Supplementary Material, Figure S1), and GPC analysis also indicated that the molecular weight of macro-CTA slightly increased from that of PCL-b-PLA, as shown in Figure 3.

Good agreement of the integral ratio between the methyl proton of TTC and the methylene proton of PCL-b-PLA (-COOCH_2_-) with that of theoretical values, along with the complete disappearance of the ω-methine group of PCL-b-PLA, are the supporting evidence that RAFT-polymerizable TTC was successfully incorporated into the PCL-PLA block copolymer. PCL-b-PLA-b-PMVK triblock copolymers were synthesized by the reaction of macro-CTA with MVK via RAFT polymerization.

**Figure 4:**
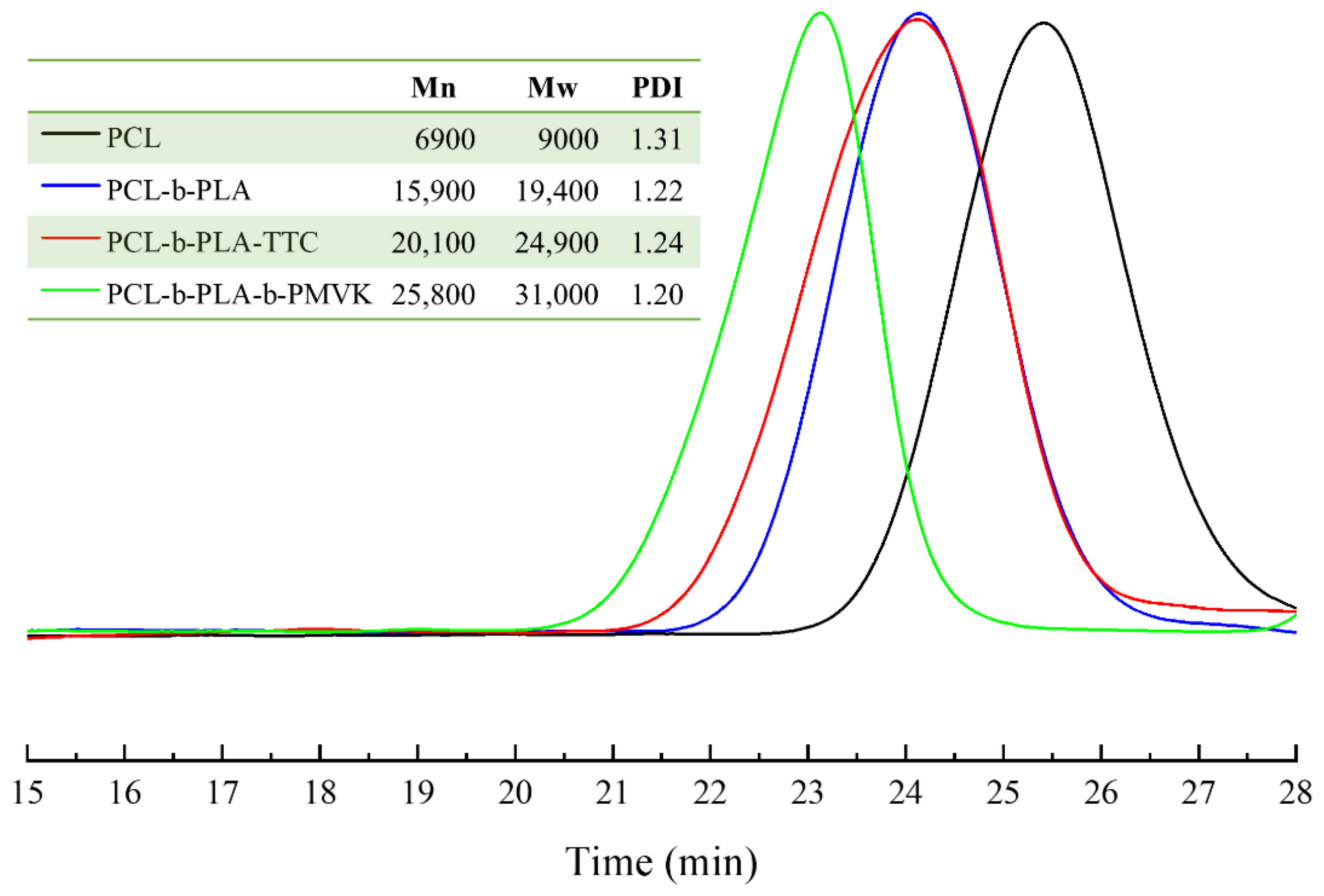
GPC traces of PCL, PCL-b-PLA, PCL-b-PLA-TTC, and PCL-b-PLA-b-PMVK (entry 2 in Table 1).

As shown in Figure 1, in the FT-IR spectrum of PCL-b-PLA-b-PMVK, stretching peaks belonging to the carbonyl groups of the PCL, PLA, and PMVK blocks were observed at 1733, 1752, and 1712 cm^−1^, providing confirmation of the formation of the triblock copolymer via RAFT polymerization. In the ^1^H NMR spectrum of PCL-b-PLA-b-PMVK (Figure 2d), protons of the methylene group (-CH_2_-CH-) of the PMVK block were found at 1.1~1.3 ppm and 1.8~2.1 ppm, while those of the methine (-CH_2_-CH-) and methyl groups in ketone were observed at 2.4 and 2.3 ppm, respectively. As shown in Figure 3, GPC traces indicated that the molecular weight and growth of PCL-b-PLA-PMVK triblock block copolymers shifted linearly to higher molecular weight as the polymerization progressed. As seen in Figure 3 and Figure 4, all GPC chromatograms showed symmetrical curves, and shifted clearly to higher elution time, while polydispersity decreased with polymerization time, supporting the occurrence of controlled polymerization.

### 3.2. Thermal Behaviors of Di- and Triblock PCL-b-PLA-b-PMVK Copolymers

**Figure 5:**
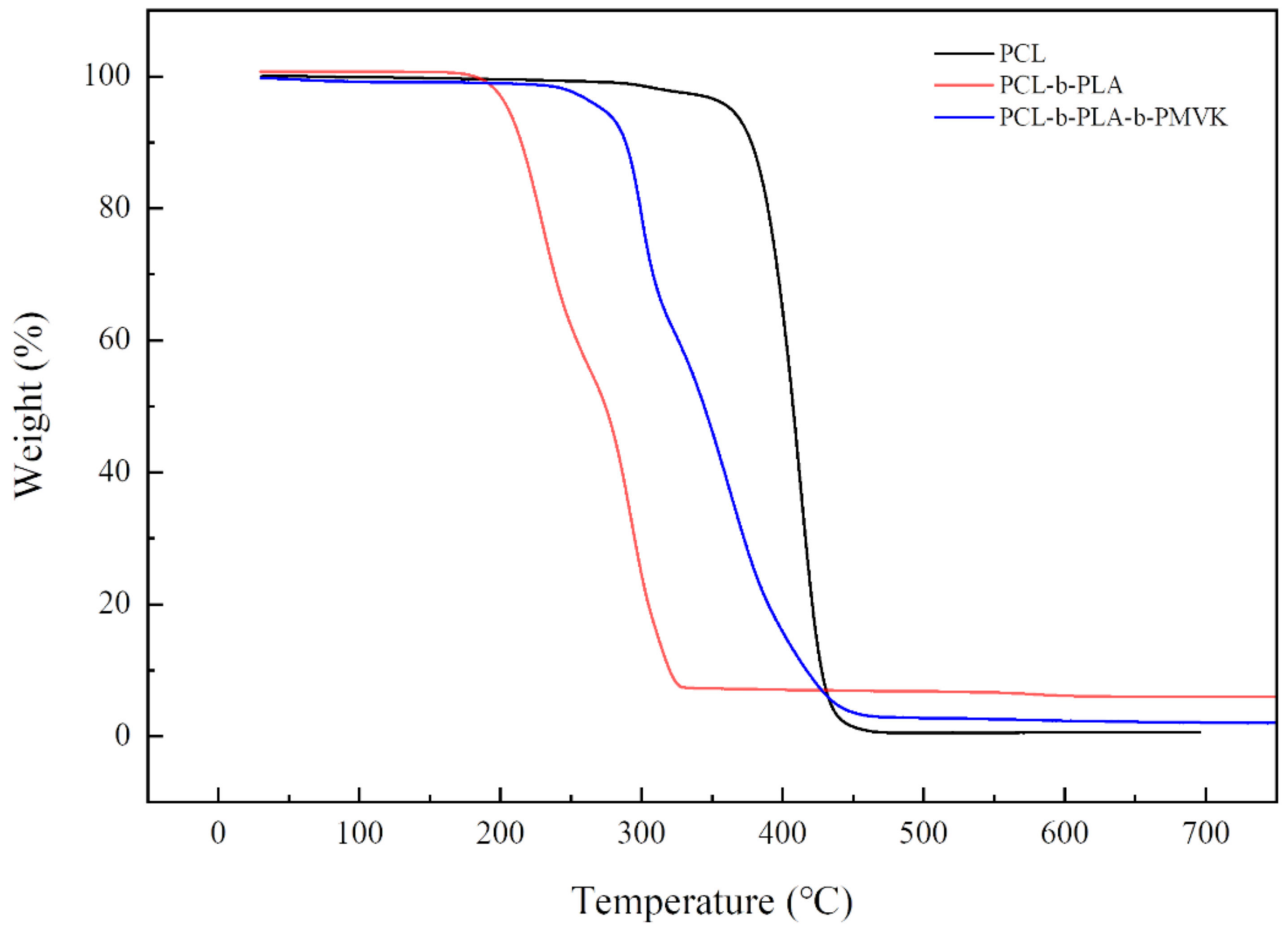
TGA thermogram of PCL, PCL-b-PLA, and the PCL-b-PLA-b-PMVK triblock copolymer.

As shown in Figure 5, compared with the thermal decomposition temperature of PCL—with initial and complete decomposition temperatures of 400 and 450 °C, respectively—the PCL-b-PLA diblock copolymer has lower initial and complete temperatures, down to 230 and 310 °C, respectively, which are also lower than those of PLA, at 310 and 410 °C, respectively [60]. This result may be attributed to the decreased crystallinity of the diblock copolymer with 50 wt% of the PCL block. Similar findings were also reported in the study of Chin et al., who prepared blends of PLA with various polymers—such as corn starch (CS), polystyrene, polyethylene, polypropylene, and polyethylene oxide—and indicated that the thermal decomposition temperature of the PLA/CS blend decreased by as much as 50% with the increase in CS content from 0 to 40 wt% [60].

**Figure 6:**
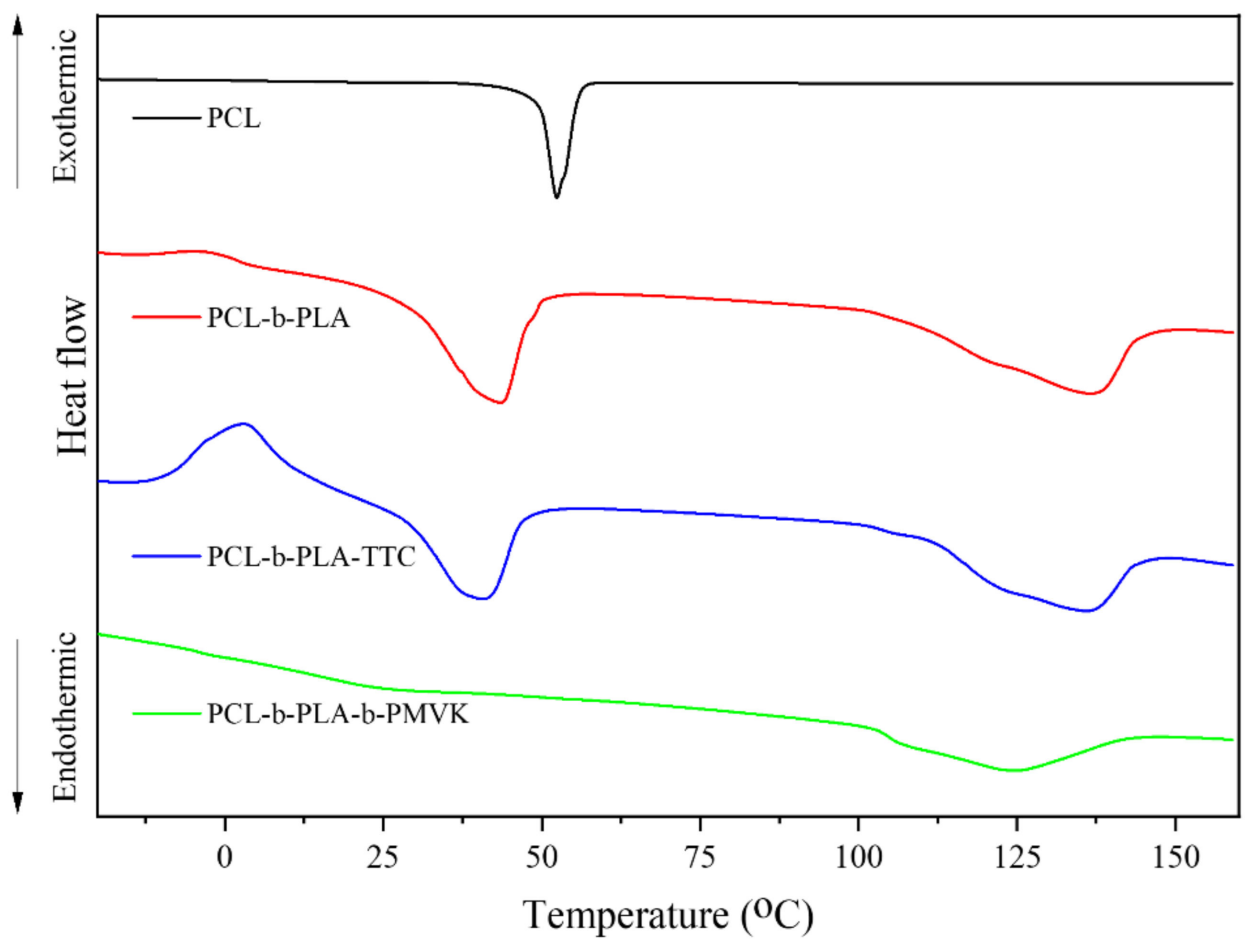
DSC thermogram of PCL, PCL-b-PLA, PCL-b-PLA-TTC, and PCL-b-PLA-b-PMVK.

More evidence of decreasing crystallinity in the block copolymers was found in the DSC curve (Figure 6). The melting temperatures of the PCL-b-PLA block copolymer were found at 42.5 and 138.5 °C, which are lower than those of the homopolymers—48 and 168 °C for PCL and PLA [61], respectively—and showed a broader range, with a decreased melting enthalpy of PCL (ΔH_m_) (Figure S2). The thermal decomposition of PCL-b-PLA-b-PMVK occurred between 320 and 410 °C (Figure 5), which are higher temperatures compared to PCL-b-PLA, due to the incorporation of the PMVK block. As shown in Figure 6 and Figure S2, the melting temperature and crystallinity of PCL-b-PLA-b-PMVK decreased compared to the PCL homopolymer and its block copolymer with PLA. As the number of blocks in the block copolymer increased, the melting and crystallization peaks became smaller, and those of PCL in the triblock copolymer nearly disappeared in the DSC thermogram, indicating that the incorporation of the PMVK block led to a decrease in the crystallinity of the triblock copolymer.

### 3.3. Photodegradation Behavior of PCL-b-PLA-b-PMVK

**Figure 7:**
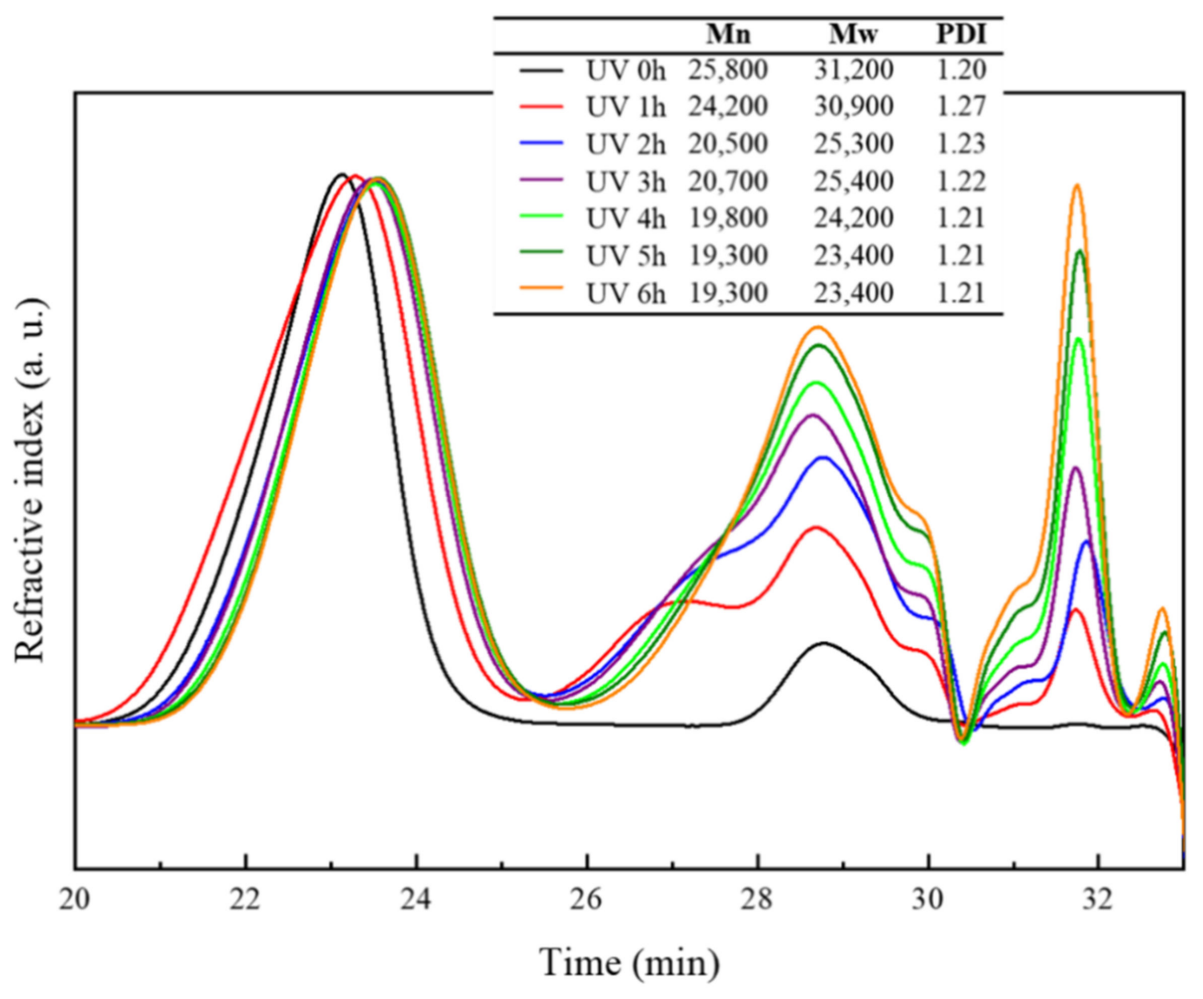
Molecular weight loss of PCL-b-PLA-b-PMVK according to UV irradiation time (entry 2 in Table 1).

After PCL-b-PLA-b-PMVK triblock copolymers were successfully synthesized via both ring-opening and RAFT polymerization, their photodegradation behavior under UV light at 1700~2000 μm/cm^2^ was observed after dissolution in THF, and was determined by the decrease in molecular weight obtained from GPC analysis. It is commonly accepted that PMVK can be degraded by two photodegradation mechanisms: radical or non-radical reactions—known as Norrish type I and Norrish type II routes, respectively—and the Norrish type II route is the main one [48]. As shown in Figure 7, the molecular weight of the triblock copolymer decreased with UV irradiation time, reaching that of the macro-CTA PCLA-DDMAT, which means that most of the PMVK block was photodegraded after 6 h of UV irradiation, together with the production of low-molecular-weight degraded residues. These results are consistent with our previous study on the synthesis and photodegradation behaviors of PCL-PMVK block copolymers [35]. GPC analysis also indicated that the amount of photodegraded residues produced, and their molecular weight, decreased with UV irradiation time.

### 3.4. Fabrication of Porous Microspheres

**Figure 8:**
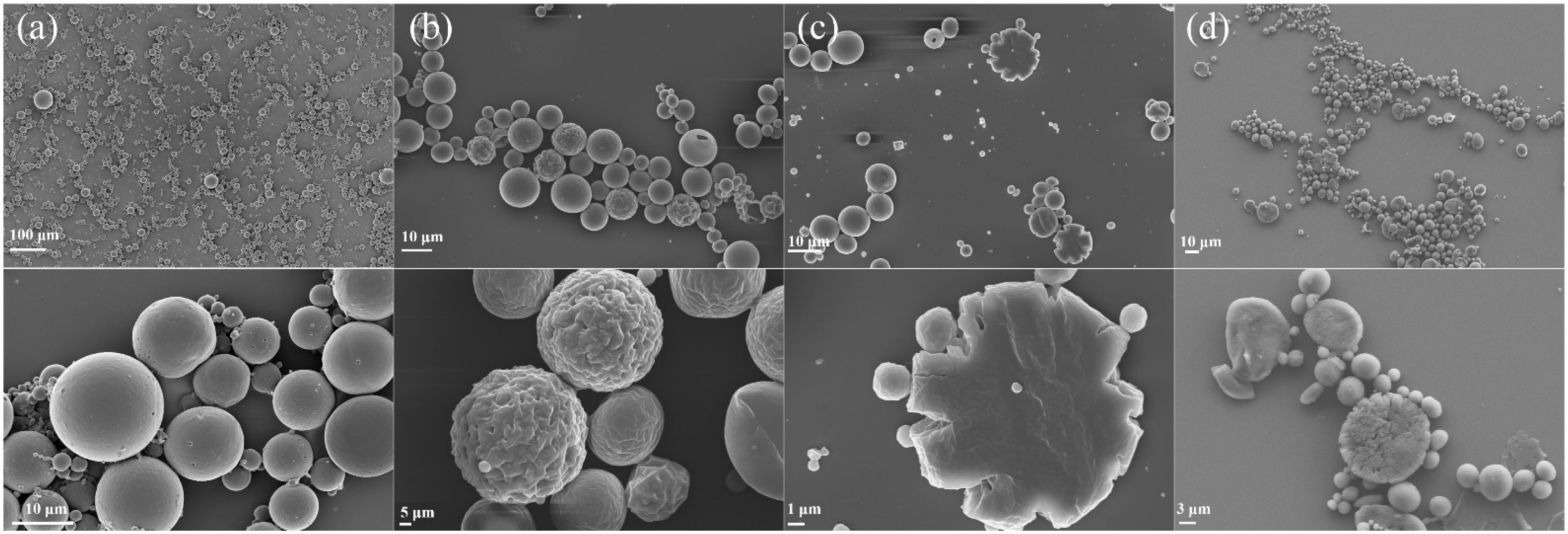
FE-SEM images of microspheres using PCL-b-PLA-b-PMVK (entry 2) according to UV irradiation time: (**a**) 0 h; (**b**) 2 h; (**c**) 4 h; (**d**) 6 h.

Various etching reagents and conditions were used for the removal of sacrificial components, including NaOH, UV light, and acidic agents. Porous microspheres based on the PCL-b-PLA-b-PMVK triblock copolymer were successfully fabricated via an oil/water (O/W) single emulsion–evaporation method, using polyvinyl alcohol as an emulsifier, followed by UV irradiation to sacrifice the photodegradable PMVK blocks. As shown in Figure 8 and Figure S3, the size of the microspheres was found to be approximately 1 μm when fabricated with 6% PVA as an emulsifier and a stirring rate of 600 rpm. The microspheres were spherical, and had smooth surfaces, before UV irradiation; however, the microspheres developed rough surfaces and porous structures after UV irradiation, due to the sacrifice of the photodegradable PMVK blocks with UV light as a porous template. In addition, for microspheres larger than 10 μm in diameter, the shapes were also changed from microspherical to flat disk shapes with increasing UV irradiation time, due to the collapse of the PMVK blocks by UV light, leading to decreased shape stability with high porosity, as reported in our previous paper [35].

**Figure 9:**
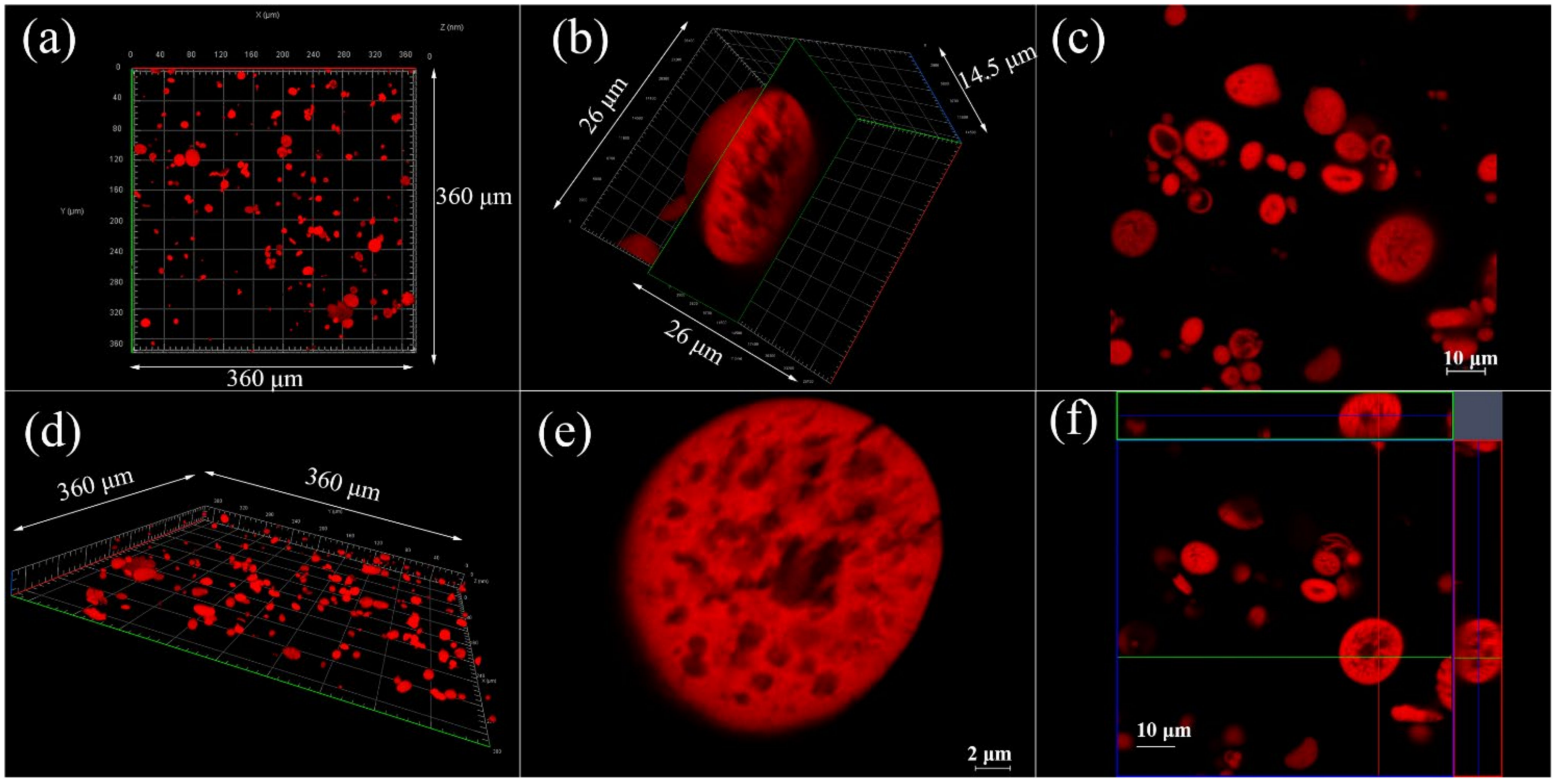
Confocal microscopy images of PCL-b-PLA porous microspheres: (**a**) top view; (**b**) internal view; (**c**) single view; (**d**) 3D view; (**e**) slice view; and (**f**) ortho view.

Confocal laser microscopy images taken by Z-stack, with a slice thickness of 0.2 μm, also showed internal porosity in the porous microspheres (Figure 9), as can be clearly observed in the three-dimensional confocal laser microscopy videos, provided as supporting ESI information. 

## 4. Conclusions

Biodegradable triblock copolymers were successfully synthesized via ring-opening polymerization of L-lactide onto PCL, followed by RAFT polymerization of methyl vinyl ketone (MVK), and used to fabricate porous, biodegradable microspheres via the oil/water single emulsion–evaporation method and photodegradation of PMVK blocks by UV irradiation at a wavelength of 312 nm. The linear relationship between molecular weight and polymerization time, along with the narrow polydispersity of the resulting PMVK initiated by PCL-b-PLA-based macro-CTA, are clear indicators of a controlled RAFT polymerization mechanism. The photodegradability of the PMVK block was proven by experiments showing both a decrease in the molecular weight of the triblock copolymers and the formation of porosity in the microspheres after UV irradiation. On the basis of these results, porous biodegradable microspheres can be considered to be potential matrices for controlled drug release, because of their high porosity and nontoxic biodegradation behavior.

## Figures and Tables

**Table 1:** Fabrication of porous microspheres based on PCL-b-PLA-b-PMVK.

Entry	PCL		PCL [mmol]	L-lactide [mmol]	MVK [mmol]	Molecular Weight				
	Type	Molecular Weight				PCL-b-PLA		PCL-b-PLA-TTC	PCL-b-PLA-b-PMVK	
						GPC	^1^H NMR	GPC	GPC	^1^H NMR
1	4arm	1000	1.2	35	200	11,700	7500	21,600	58,500	34,300
						(1.13)		(1.22)	(1.46)	
2	Diol	4000	5	140	50	15,900	8100	20,100	25,800	12,700
						(1.22)		(1.24)	(1.20)	
3	Diol	4000	5	75	25	7500	5800	8800	10,600	6900
						(1.17)		(1.24)	(1.22)	
4	Diol	4000	5	75	50	7500	5800	8800	12,300	8200
						(1.17)		(1.24)	(1.22)	

## Data Availability

Not applicable.

## Supplementary Materials

The following are available online at https://www.mdpi.com/article/10.3390/polym13223964/s1: Additional experimental details and characterization data (DOCX): ^13^C NMR, DSCm and DLS. Four additional videos of PCL-b-PLA porous microspheres (AVI). Figure S1: 13C NMR spectrum of PCL-b-PLA-TTC. Figure S2: DSC cooling curve of PCL, PCL-b-PLA, PCL-b-PLA-b-PMVK. Figure S3: DLS measurements of PCL-b-PLA-b-PMVK microspheres.
