# Supplementary material for: Biodegradable PCL-b-PLA Microspheres with Nanopores Prepared via RAFT Polymerization and UV Photodegradation of Poly(Methyl Vinyl Ketone) Blocks
Source: Polymers (Basel). 2021 Nov 16;13(22):3964. doi: 10.3390/polym13223964 (PMC8622187; doi:10.3390/polym13223964)
Supplement: Supplementary file 1 [file polymers-13-03964-s001.zip › Polymers_Supplementry Data (Ildoo Chung).pdf]

Supplementary data for

Biodegradable PCL-b-PLA Microspheres with Nanopores Prepared by RAFT Polymerization and UV Photodegradation of Poly(methyl vinyl ketone) Blocks

Taeyoon Kim[1,2], Sorim Lee[1], Soo-Yong Park[1], Ildoo Chung[1,*]

[1] *Department of Polymer Science and Engineering, Pusan National University, Busan 46241, Korea*

[2] *Convergence Research Center for Energy and Environmental Sciences, Sungkyunkwan University (SKKU), Suwon 16419, Korea*

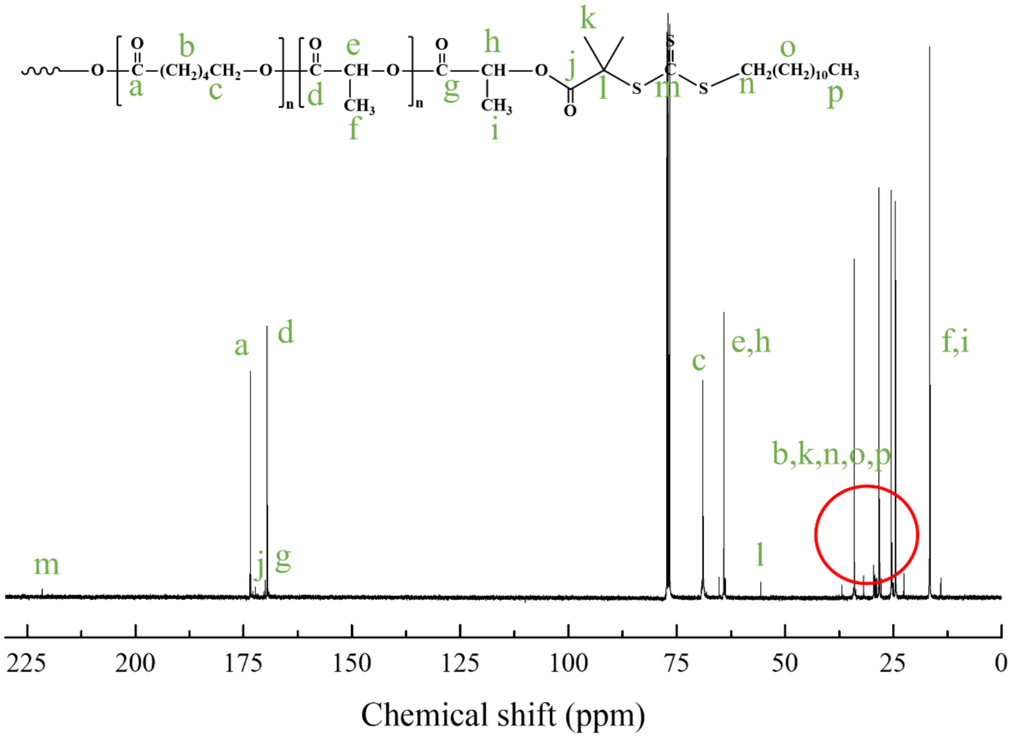

Figure S1. ^{13}C NMR spectrum of PCL-b-PLA-TTC.

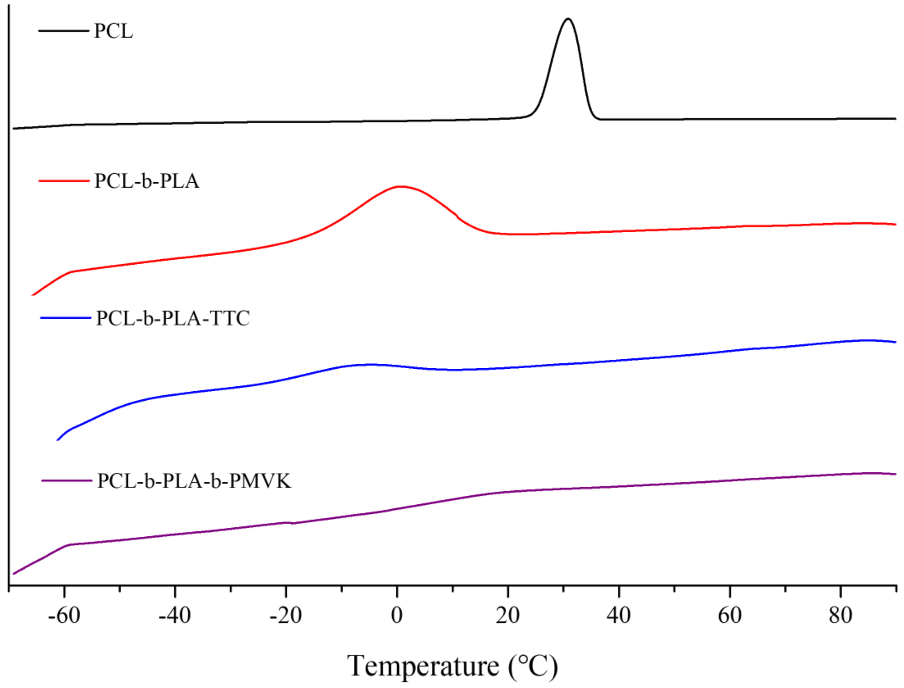

Figure S2. DSC cooling curve of PCL, PCL-b-PLA, PCL-b-PLA-b-PMVK.

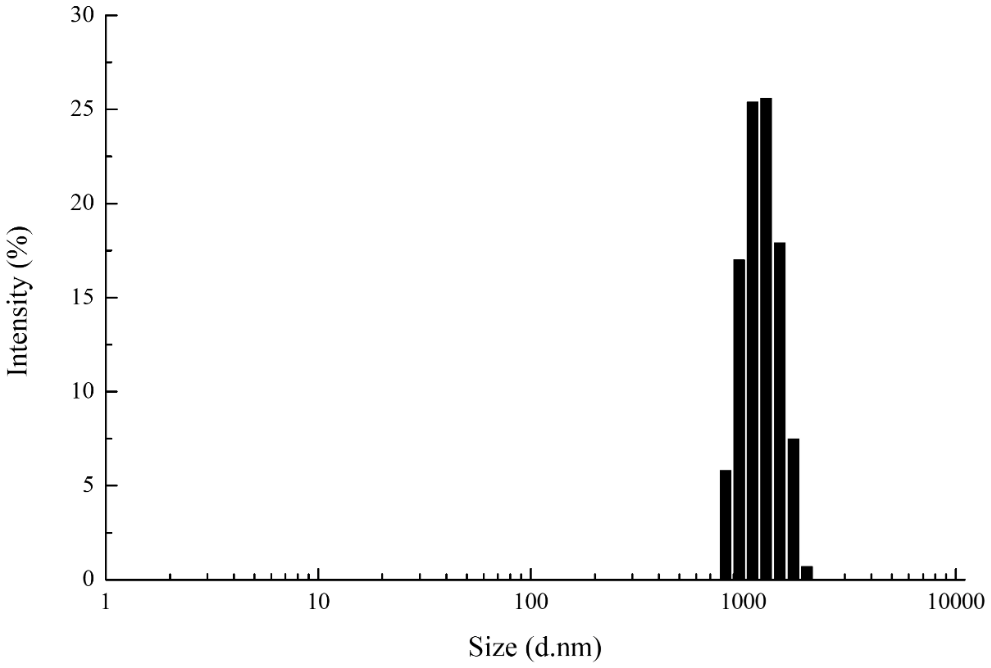

Figure S3. DLS measurements of PCL-b-PLA-b-PMVK microspheres.
